# MIGRENE: The Toolbox for Microbial and Individualized GEMs, Reactobiome and Community Network Modelling

**DOI:** 10.3390/metabo14030132

**Published:** 2024-02-21

**Authors:** Gholamreza Bidkhori, Saeed Shoaie

**Affiliations:** 1 Centre for Host–Microbiome Interactions, Faculty of Dentistry, Oral & Craniofacial Sciences, King’s College London, London SE1 9RT, UK; gholamreza.bidhkori@kcl.ac.uk; 2 Science for Life Laboratory, KTH—Royal Institute of Technology, 171 21 Stockholm, Sweden

**Keywords:** microbiome, metabolism, genome scale metabolic model, host–microbiome interaction

## Abstract

Understanding microbial metabolism is crucial for evaluating shifts in human host–microbiome interactions during periods of health and disease. However, the primary hurdle in the realm of constraint-based modeling and genome-scale metabolic models (GEMs) pertaining to host–microbiome interactions lays in the efficient utilization of metagenomic data for constructing GEMs that encompass unexplored and uncharacterized genomes. Challenges persist in effectively employing metagenomic data to address individualized microbial metabolisms to investigate host–microbiome interactions. To tackle this issue, we have created a computational framework designed for personalized microbiome metabolisms. This framework takes into account factors such as microbiome composition, metagenomic species profiles and microbial gene catalogues. Subsequently, it generates GEMs at the microbial level and individualized microbiome metabolisms, including reaction richness, reaction abundance, reactobiome, individualized reaction set enrichment (iRSE), and community models. Using the toolbox, our findings revealed a significant reduction in both reaction richness and GEM richness in individuals with liver cirrhosis. The study highlighted a potential link between the gut microbiota and liver cirrhosis, i.e., increased level of LPS, ammonia production and tyrosine metabolism on liver cirrhosis, emphasizing the importance of microbiome-related factors in liver health.

## 1. Introduction

Constraint-based modeling, utilizing genome-scale metabolic models (GEMs), offers a mechanistic insight into microbial metabolism and genotype–phenotype correlations within host–microbiome interactions [1]. GEMs have been employed for rudimentary microbial community modeling within the gut, showcasing their capacity to discern the pivotal metabolic contributions of bacteria in an ecosystem to the host’s metabolism. Previously, multiple GEMs of human gut bacteria have been formulated, predominantly relying on available whole-genome sequencing data. These models have been instrumental in exploring bacterial growth rates under varying nutrient availability on a community level [2,3], such as AGORA2, an expanded resource of human gut microbial metabolic reconstructions incorporating 7302 strains [4]. To facilitate the automated reconstruction of microbial GEMs, web-servers like KBase [5] and ModelSEED [6] integrate genome sequences and metagenomic datasets. In the last decade, many computational tools have been developed to design and simulate microbial communities, under either steady-state (MICOM [7], SteadyCom [8], cFBA [9]), dynamic (dyMMM [10], DAPHNE [11], surfinFBA [12]), or spatiotemporal conditions (COMETS [13], BacArena [14] CROMICS [15]). Steady-state approaches are suitable for describing growth in CSBR (chemostats or continuous stir batch reactor) systems. Tools based on dynamic approaches are suitable for describing non-continuous systems and some continuous systems. The spatiotemporal tools aim to describe 2D dimensional surface environments, such as mimicking simple solid-state Petri dish environments [16].

Despite this advancement, the hurdle remains in effectively applying metagenomic data for both individual-level microbial metabolisms and community-level metabolic modeling. A significant challenge endures, specifically in the capability to directly harness metagenomic data for the reconstruction of GEMs that encompass uncultured and not yet characterized genomes, subsequently employing these models for personalized microbiome investigation. Zorrilla et al. introduced metaGEM for GEM reconstruction from metagenome assembled genomes (MAGs) and the subsequent predictions of interactions between microbes in samples [17]. Furthermore, 247,092 genome-scale metabolic modes were built from 92,143 MAGs [18] and 154,723 MAGs [19].

The exploration of metagenomic gene content involves the creation of “gene catalogs”, which comprise all genes identified through assembly across many samples. In the context of the human gut microbiome, a catalog containing 10 million non-redundant genes (named IGC2) was built using de novo methods [20]. Recently, comprehensive microbial gene catalogs have been unveiled, encompassing 45,666,334 non-redundant genes for gut and oral samples [21], along with the non-redundant gene catalog specific to the human vaginal microbiome, known as VIRGO [22]. Employing the latest development of integrated non-redundant microbial gene catalogues and metagenome binning to acquire high-quality assembled genomes represents a comprehensive approach to obtaining high-quality GEMs directly from metagenomic data. Here, we present the toolbox for microbial and individualized GEMs, reactobiome and community network modelling (MIGRENE), which enables the generation of species and community-level models from any reference gene catalogues and/or metagenome species to be applied to personalized microbiome studies. As an application, we then utilized the toolbox to investigate reaction richness and GEM richness, as well as the reactobiome in individuals with liver cirrhosis.

## 2. Method

**Figure 1:**
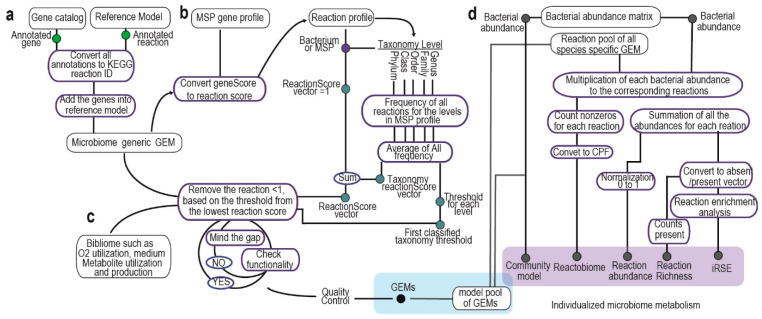
MIGRENE toolbox workflow. (**a**) Integration of bacterial gene catalog into metabolic model and microbiome reference GEM generation. (**b**,**c**) Calculation of reaction score and species-specific model generation. (**d**) Generating individualized microbiome metabolism.

The MIGRENE toolbox effectively utilizes any non-redundant microbial gene catalogue and metagenome species pangenomes (MSPs) to create species-specific Genome-Scale Metabolic Models (GEMs). The initial phase of the workflow involves generating a generalized microbiome GEM through the integration of the non-redundant microbial gene catalogue and a metabolic model (Figure 1a). The reaction profile and reaction score for each metagenome species are established by mapping to the reference model and utilizing taxonomical information (Figure 1b). Subsequently, species-specific GEMs are reconstructed based on the reaction score and bibliomic data (Figure 1c). The analytical component of the toolbox operates using these GEM models, and it’s also possible to incorporate other microbial GEMs into the metagenomics data to enable “individualized metabolic microbiome analysis”. This process generates five distinct outputs at an individualized level: reactobiome, reaction abundance, reaction richness, community modeling and individualized reaction set enrichment (iRSE) (Figure 1d).

### 2.1. Integration of Bacterial Gene Catalog into Metabolic Model and Microbiome Reference GEM Generation

First, the bacterial gene catalogue undergoes restructuring and verification using the “checkCatalog.m” function. The “convertCatalogAnnotation.m” function is then employed to translate KO annotations from the catalogue into KEGG reaction IDs. If the mapping ID file is not specified as an input argument, the function automatically fetches the required conversion information from the KEGG API and stores it in the “data” directory within the MIGRENE Toolbox location.

Next, a comprehensive annotated metabolic model is generated by leveraging the resources of the generic metabolic model (such as ModelSEED and KBase). Both the generic metabolic model and the gene catalog serve as inputs for the “microbiomeGEMgeneration.m” function, facilitating the creation of a reference genome-scale metabolic model (GEM). The association between genes, proteins and reactions (Gene–Protein–Reaction or GPR) is established by integrating the catalog genes into the metabolic model using KEGG reaction IDs. In cases where the GEM’s annotation file is not provided, the function autonomously identifies annotations such as KO, reaction ID or EC within the model and converts them into reaction IDs based on the most recent version of KEGG IDs. Additional fields, namely “grRules”, “genes”, “rules”, “geneNames”, and “rxnGeneMat”, are introduced into the GEM to ensure compatibility with both COBRA and RAVEN GEMs. Consequently, the resulting models can be seamlessly employed with the functions offered by both toolboxes.

### 2.2. Calculation of Reaction Score and Taxonomy Threshold

The annotated reference GEM was used to convert the MSP gene profile to reaction profiles. The reaction states (absent/present) for each MSP were generated based on the presence of MSP genes in the corresponding gene rule vector of the reaction in the reference GEM. A matrix of reaction frequency was also calculated using the average of frequency of each reaction in the taxonomy levels (i.e., genus, family, order, class, phylum) of the corresponding MSP. Reaction scores for each MSP were calculated by adding the reaction state and reaction frequency matrices entries together, and, as a result, the reaction score profiles for the MSPs were made. A threshold (the lowest non-zero frequency) was determined for each taxonomy level for filtering the reactions while generating the species-specific GEMs (pseudocode is available in Supplementary Notes). The MetagenomeToReactions.m function creates a reaction, and the reaction states for all the MSPs and taxonomy information are collected using GenerateMSPInformation.m. MetaGenomicsReactionScore.m calculates reaction scores for each MSP (bacterial species).

### 2.3. Species-Specific Genome-Scale Metabolic Model Generation

To create species-specific metabolic models, a process is followed using the following functions. The initial step involves constraining a general gut reference Genome-Scale Metabolic Model (GEM) based on one of the available diets in the toolbox. This is accomplished using the “DietConstrain.m” function. Five distinct diets are provided: high-protein plant-based, high-protein omnivorous, high-fiber plant-based, high-fiber omnivorous and an average UK diet. These diets are formulated based on their macronutrient composition. Each diet is essentially an average daily meal plan, spanning three main meals and a snack. The ingredients in these meals are quite similar across the diets, with some substitutions made to align with the specific dietary requirements. The composition of each diet is specified in appropriate portion sizes and normalized to a 2000 kCal daily intake. Acetate and Lactate are introduced as popular carbon sources for bacteria to enable constraint-based modeling.

The subsequent steps involve using two key functions, “contextSpecificModelGeneration.m” and “contextSpecificModelTune.m”, to generate and refine the GEM models. Rather than a traditional gap-filling approach, the former function prunes the constrained reference GEM starting from the lowest reaction score and fills in the gaps strategically to maintain the model’s functionality based on the biomass objective function and a predefined threshold. The gap-filling process begins with reactions that have the highest frequency in the closest taxonomy level of the target species.

The “contextSpecificModelTune.m” function fine-tunes the models further. It prunes exchange and transport reactions, addresses dead-end metabolites, adds compartments to the model, updates the model description and corrects KEGG metabolite IDs and metabolite formulas. Additionally, it calculates essential information, such as the gap-filling percentage at different levels, which includes taxonomy proximity-based gap filling, gap filling at further taxonomy levels, handling reactions without gene–protein–reaction relations, and the total gap-filling percentage.

The growth rates for each model can be assessed under different diet conditions using constraint-based modeling. The toolbox also provides valuable data on the structural characteristics, connectivity, and carbon balance of the generated GEMs. This comprehensive approach allows for the creation of species-specific metabolic models tailored to specific diets and offers insights into how these models function in response to different dietary conditions.

### 2.4. Generation of Individualized Metabolic Microbiome: Reaction Richness, Reaction Abundance and Reactobiome, iRSE and Community Models

We utilize a reaction pool from GEMs and the abundance of Metagenome Species (MSP) to create personalized microbiome metabolism. Reaction richness profiles, representing gut microbiome compositions, are computed using the RxnRichnessGenerator.m function. Each MSP’s abundance contributes to the reactions of the sample, forming a matrix (n × m; n: reaction pool size, m: MSP count) for each sample. This matrix becomes a binary vector, indicating the presence/absence of reactions in samples, capturing reactions present in at least one MSP (Supplementary Figure S1). The ReactionAbundanceGenerator.m sums reaction abundances to yield relative reaction abundance profiles. Reactobiome represents normalized reactions per 500 bacteria (CPF) in the gut for each individual, calculated using CPFGenerator.m. Individualized reaction set enrichment (iRSE) is generated by pRSEGenerator.m, showing overrepresentation of 153 KEGG metabolic pathways (provided in the toolbox) in samples based on a hypergeometric test and a binary vector.

Community models are created for each sample using MakeCommunity.m. Bacterial species names are added as prefixes to reaction names in GEMs, segregating species-specific GEMs in the combined S matrix. Compartments [lu] (intestinal lumen) and [fe] (secreted bacterial and food-derived metabolites) are included and connected by exchange reactions. [lu] represents the gut microbiome with bacterial GEMs and exchange reactions for metabolites. Community biomass objective function accounts for bacterial biomass, with bacterial abundance as stoichiometric coefficients.

### 2.5. Processing and Downstream Analysis of Liver Cirrhosis Metagenomics

Publicly available liver cirrhosis shotgun metagenomics data [23] were retrieved from European Nucleotide Archive (ENA) repository under the identifier ERP005860. Samples with a read depth below 10 million reads were excluded. Utilizing METEOR, a quantitative metagenomic profiling software [24], along with the gut microbial gene catalog [20], quality control, trimming, and mapping were conducted to generate gene count tables. To enhance precision and consistency, downsizing was employed using a threshold of 10 million. Following this, MetaOMineR [25] was applied to normalize gene counts, ensuring accurate representation of metagenome species (MSP) abundance.

## 3. Results

To assess the functional role of the individualized gut microbiota in liver cirrhosis, we computed various metrics, including reactobiome, reaction abundances, reaction richness, and GEM (Genome-Scale Metabolic Model) richness for each individual, utilizing 1333 MAGMA GEM models available at www.microbiomeatlas.com.

**Figure 2:**
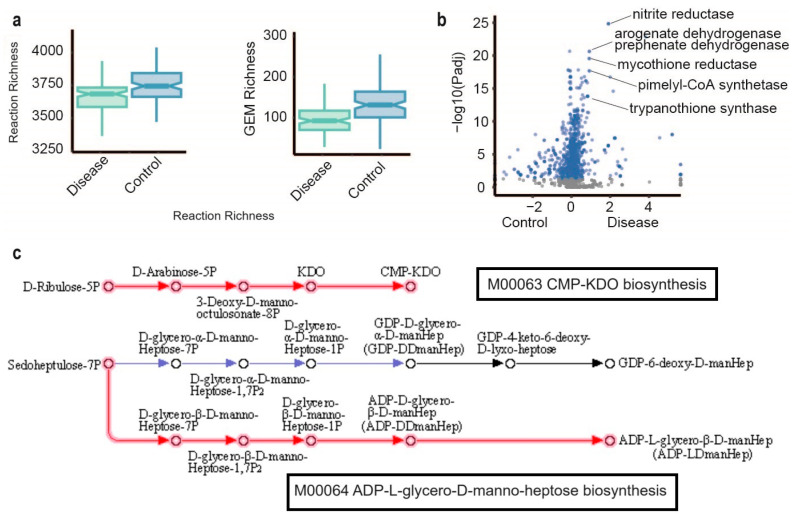
Microbiome metabolic features in gut microbial liver cirrhosis. (**a**) The boxplot of reaction richness and GEM richness in disease and control gut microbial samples. (**b**) Reactions enriched (shown in blue) in disease relative to control samples at FDR 0.01, Wilcoxon test. (**c**) Enriched reactions (red arrows) in disease compared to control samples in KEGG modules M00063 and M00064 at FDR 0.01, Wilcoxon test.

In this study, we demonstrated a significant reduction in both reaction richness and GEM richness in individuals with liver cirrhosis (Supplementary Table S1, Figure 2a). When comparing the reactobiome between disease and control gut microbial samples, it was observed that a total of 822 reactions exhibited significant differences (as determined by the Wilcoxon signed-rank test with a false discovery rate of less than 1 × 10^−5^, as presented in Supplementary Table S2, Figure 2b). These reactions included notable enzymes, such as nitrite reductase, arogenate dehydrogenase, prephenate dehydrogenase, mycothione reductase, pimelyl-CoA synthetase, and trypanothione synthase. Notably, nitrite reductase emerged as the most enriched reaction in liver cirrhosis gut microbial samples, with a false discovery rate of 1.45^−25^.

Among the significantly enriched reactions in liver cirrhosis, 21 reactions, identified by their KEGG reaction IDs (refer to Supplementary Table S3 and Supplementary Figure S2), were linked to tyrosine metabolism. Liver insufficiency has been associated with alterations in plasma amino acid concentrations, with phenylalanine and tyrosine levels increasing with the severity of liver disease [26]. Intriguingly, Sato et al. demonstrated that elevated serum tyrosine concentration may serve as an indicator of a high risk of death or liver transplantation for patients with liver cirrhosis [27]. These findings suggest that tyrosine metabolism within the gut microbiota could exemplify a host–microbe interaction.

The reactions of two KEGG modules M00063 (CMP-KDO biosynthesis) and M00064 (ADP-L-glycero-D-manno-heptose biosynthesis) were significantly enriched in liver cirrhosis gut microbial samples. The sugars CMP-KDO (3-deoxy-d-manno-oct-2-ulosonic acid (Kdo)) and ADP-L-glycero-D-manno-heptose biosynthesis are components of bacterial lipopolysaccharide (LPS) (Figure 2c). The studies showed that elevated levels of serum LPS are linked to an increased risk of hospitalization, cancer or mortality due to liver disease in the general population.

## 4. Discussion

We have developed the MIGRENE Toolbox, a comprehensive platform designed to seamlessly integrate microbial gene catalogues, metagenomic species data and general metabolic models. This integration empowers the generation of Genome-Scale Metabolic Models (GEMs) and community-level models, facilitating their application in individualized microbiome studies.

Recent studies have showcased the remarkable power of the MIGRENE Toolbox in unravelling the relationships between the human microbiome and various metabolic disorders. One such notable application involved investigating the mechanistic implications of the gut microbiome in three distinct metabolic disorders: obesity, type 2 diabetes and atherosclerosis. By harnessing the capabilities of the MIGRENE Toolbox, Proffitt et al. uncovered commonalities among these conditions, observing a shared phenomenon characterized by an elevated glutamate consumption by gut bacteria concomitant with the production of ammonia, arginine and proline [28].

Furthermore, the toolbox’s was utilized in a study by Ezzamouri et al. [29]. They showed an association between the human gut microbiome and type 2 diabetes mellitus. Delving even deeper, they explored the intricate changes in bacterial metabolism that occur following metformin treatment, a cornerstone in the management of diabetes.

The MIGRENE Toolbox was instrumental in elucidating the microbial landscape in Parkinson’s disease (PD). Rosario et al. uncovered a heightened microbial capacity for mucin and host glycan degradation within the context of PD. Additionally, they identified a deficiency in folate production by the gut microbiota, potentially linked to the development and progression of this neurodegenerative disease [30]. This groundbreaking insight into the role of the microbiome in PD offers new possibilities for understanding the disease’s pathophysiology.

In this study, we revealed a significant reduction in both reaction richness and GEM richness in individuals with liver cirrhosis. This finding is in line with previous research that has indicated a similar loss of gene richness through various health conditions, including obesity and Inflammatory Bowel Disease (IBD) [25,31,32]. Notably, our investigation is closely aligned with the observations made in the liver cirrhosis cohort, where the gene richness decreased in the disease gut microbial samples [23].

Our results showed a potential link between the gut microbiota and hepatic encephalopathy due to enrichment in ammonia production and tyrosine metabolism, which could play a role in the host–microbe interaction in liver cirrhosis. The enrichment of modules associated with ammonia production in patients could suggest a potential involvement of the gut microbiota in hepatic encephalopathy, a complication linked to liver cirrhosis characterized by elevated ammonia levels. Excessive ammonia production by gut bacteria may contribute to elevated blood ammonia levels [23]. Additionally, the study highlights enrichment of LPS production reactions in liver cirrhosis, emphasizing the importance of microbiome-related factors in liver health. Mannisto et al. showed that individuals in the highest tertile of serum LPS levels may contribute to as much as 30% of the overall risk of developing liver disease [33].

## 5. Conclusions

In summary, the MIGRENE Toolbox represents a transformative tool in microbiome research, enabling the construction of intricate models and offering a deeper understanding of the intricate relationships between the microbiome and metabolic disorders. Its versatility and power have been showcased in various studies, from uncovering shared metabolic patterns across different diseases to elucidating the role of the microbiome in diabetes and neurodegenerative conditions. With its continued development and application, the MIGRENE Toolbox promises to unlock new frontiers in microbiome research, personalized medicine and disease management.

The absence of a comprehensive list of metagenome species and the limited availability of whole genome data for identified microbes may constrain functional annotation and metabolic model generation. This limitation could affect gap filling, bacterial metabolic capacity and listed reactions. However, the approach of utilizing various taxonomical information sources for gap filling helps mitigate these challenges. Additional limitations may arise in constraint-based analyses, especially in determining biomass as the objective function, due to the difficulty in measuring biomass for many metagenome species. Current inability to isolate or cultivate these species makes exact biomass measurements impractical, leading to the use of approximations based on other species.

## Data Availability

The MIGRENE toolbox can be found at our GitHub repository https://github.com/sysbiomelab/MIGRENE. The code for the Figure 2a,b was made available in the GitHub repository https://github.com/sysbiomelab/LiverCirrhosis_MS.

## Supplementary Materials

The following supporting information can be downloaded at: https://www.mdpi.com/article/10.3390/metabo14030132/s1, Supplementary Figure S1. Calculation of reaction abundance and richness for three individuals using three bacterial abundance and the corresponding GEMs. rxn: reaction; A/P: absent/present; Supplementary Figure S2. Tyrosine metabolism. Reactions enriched (shown in red) in liver cirrhosis relative to control samples at FDR 0.01, Wilcoxon test. Supplementary Note: Pseudocode for calculation of reaction score and taxonomy threshold. Supplementary Table S1: Liver cirrhosis cohort including sample ID, reaction richness and GEM richness. Supplementary Table S2: List of reactions involved in tyrosine metabolism significantly enriched in liver cirrhosis samples using Wilcoxon two-sided tests adjusted *p*-value and FDR. Supplementary Table S3: KEGG tyrosin metabolism reactions enriched in liver cirrhosis.
