# Supplementary figures and images for: MIGRENE: The Toolbox for Microbial and Individualized GEMs, Reactobiome and Community Network Modelling
Source: Metabolites. 2024 Feb 21;14(3):132. doi: 10.3390/metabo14030132 (PMC10972203; doi:10.3390/metabo14030132)

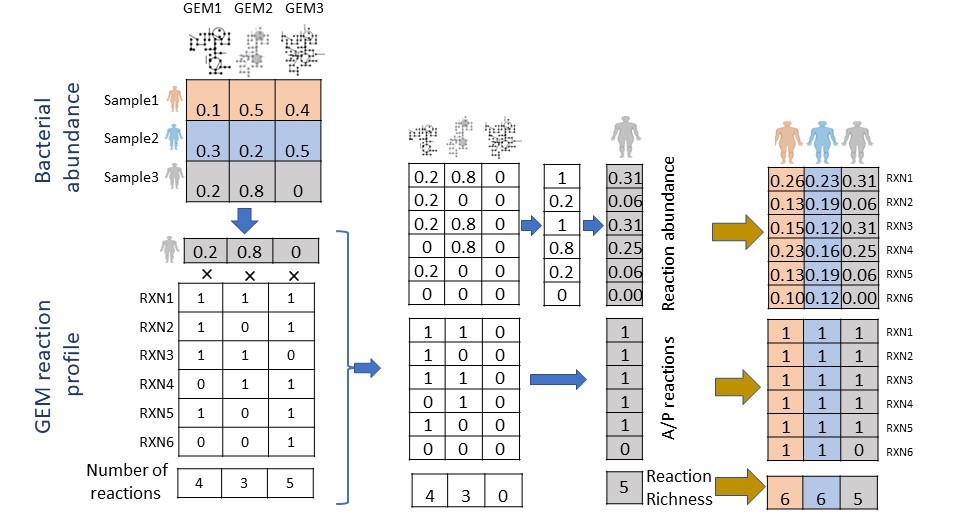

Supplement: Supplementary file 1 [file metabolites-14-00132-s001.zip › supplementary Figure S1.jpg]

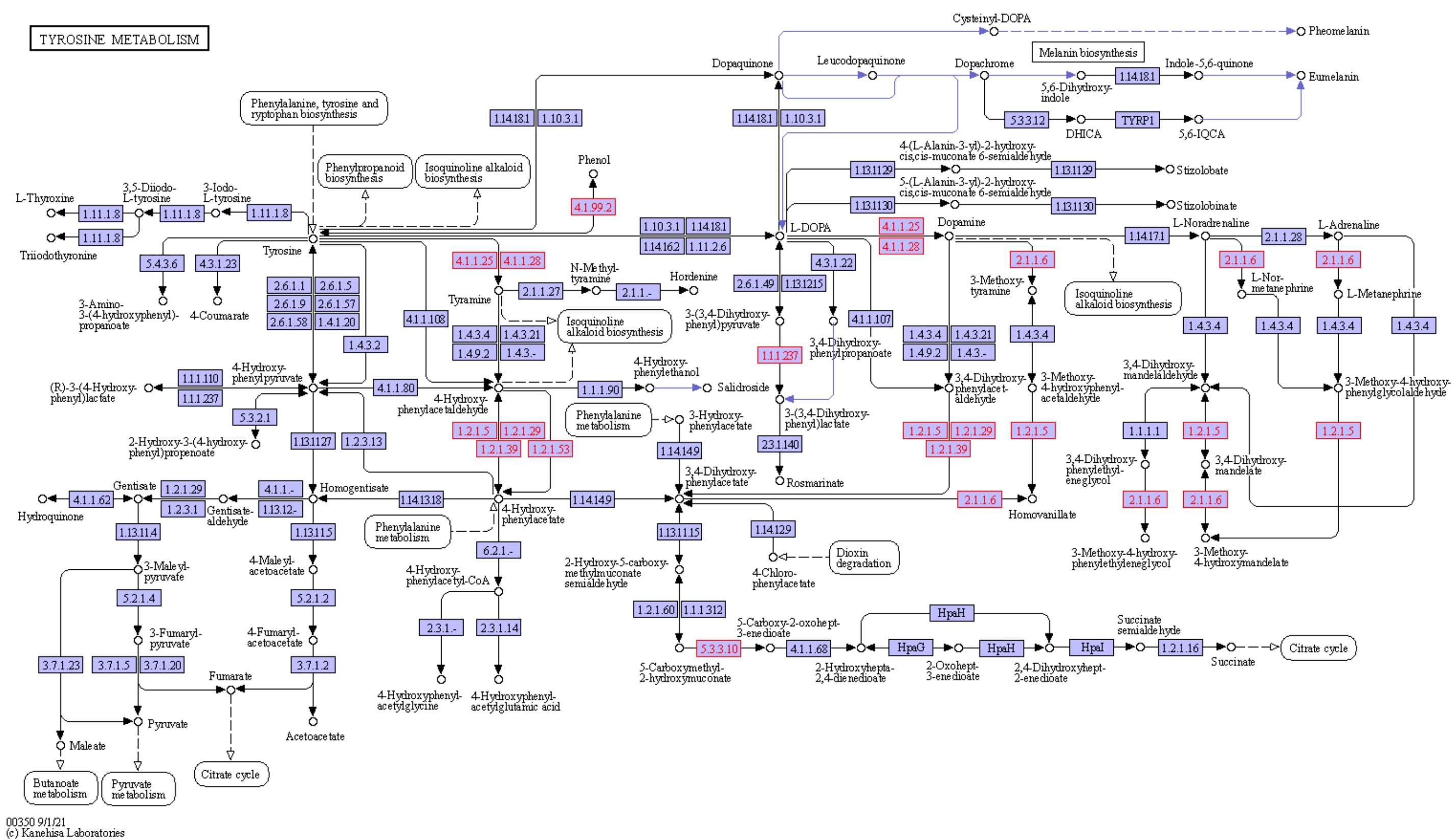

Supplement: Supplementary file 1 [file metabolites-14-00132-s001.zip › supplementary Figure S2.jpg]
